# Identification of inverse kinematic parameters in redundant systems: Towards quantification of inter-joint coordination in the human upper extremity

**DOI:** 10.1371/journal.pone.0278228

**Published:** 2022-12-16

**Authors:** Mahdi Khoramshahi, Agnes Roby-Brami, Ross Parry, Nathanaël Jarrassé

**Affiliations:** 1 Sorbonne Université, CNRS, INSERM, Institute for Intelligent Systems and Robotics (ISIR), Paris, France; 2 Laboratoire LINP2-2APS, UPL, Université Paris Nanterre, Nanterre, France; Swansea University, UNITED KINGDOM

## Abstract

Understanding and quantifying inter-joint coordination is valuable in several domains such as neurorehabilitation, robot-assisted therapy, robotic prosthetic arms, and control of supernumerary arms. Inter-joint coordination is often understood as a consistent spatiotemporal relation among kinematically redundant joints performing functional and goal-oriented movements. However, most approaches in the literature to investigate inter-joint coordination are limited to analysis of the end-point trajectory or correlation analysis of the joint rotations without considering the underlying task; e.g., creating a desirable hand movement toward a goal as in reaching motions. This work goes beyond this limitation by taking a model-based approach to quantifying inter-joint coordination. More specifically, we use the weighted pseudo-inverse of the Jacobian matrix and its associated null-space to explain the human kinematics in reaching tasks. We propose a novel algorithm to estimate such Inverse Kinematics weights from observed kinematic data. These estimated weights serve as a quantification for spatial inter-joint coordination; i.e., how costly a redundant joint is in its contribution to creating an end-effector velocity. We apply our estimation algorithm to datasets obtained from two different experiments. In the first experiment, the estimated Inverse Kinematics weights pinpoint how individuals change their Inverse Kinematics strategy when exposed to the viscous field wearing an exoskeleton. The second experiment shows how the resulting Inverse Kinematics weights can quantify a robotic prosthetic arm’s contribution (or the level of assistance).

## 1 Introduction

“*How can we measure the quality of human movement?*” Many researchers from several disciplines, especially human movement scientists, aim to answer this daunting question. One particular instance of this question concerns the redundancy of human degrees of freedom (DoF). For example, any hand movement can be generated using countless configurations of proximal joint rotations. Despite such an abundance of possible solutions, it is well known that individuals exhibit a consistent kinematic behavior; e.g., consistent use of all 7 DoF of the shoulder-elbow-wrist kinematic chain to reach and grasp an object in space [1]. Therefore, as initially put forward by Bernstein [2] the goal is to understand how joints coordinate and contribute to creating the desired hand movement. Numerous experimental approaches have sought to understand the choice of inter-joint configuration as a function of movement direction [3] velocity [4], comfort [5], fatigue [6], or task dynamics [7]. Recent theoretical approaches to human motor control have further investigated this question providing several hypotheses for the underlying control mechanism [8–11]. For instance, one prevalent hypothesis is the view that degrees of freedom are combined into synergies with automatic compensation between them [12]; i.e., neuromuscular synergy leading to “kinematic synergy” most often quantified through correlation analysis.

Despite the growing number of studies on human kinematics, there is no straightforward method of analysis to explain how kinematic redundancy is resolved. In other words, “*how can we quantify the inter-joint coordination toward a task in kinematically redundant systems?*”. Beyond fundamental investigations on human motor control, a pertinent metric is much needed in the clinics, particularly in the context of neurorehabilitation and assistive technology. Most metrics for measuring the quality of motion are limited to the end-point (or the “end-effector” as in the robotic nomenclature) and how well the task is performed. For example, in studies on post-stroke recovery, such measurement scales may identify improvements in task execution. However, no clear explanation can be provided on how the different segments (especially the dysfunctional ones) contribute to that performance. It is well known that, due to the kinematic redundancy, humans can use proximal joints to compensate for the lack of functionality of the distal ones [13, 14]. Thus, in those studies, whether the improvement is due to the actual recovery of distal joints or the compensatory role of proximal joints is ambiguous. Therefore, as argued by [15, 16], it is crucial to distinguish between kinematic compensation and restitution. However, no analysis method in the literature can explicitly address this issue [17–19]. Most metrics for inter-joint coordination are limited to correlation analysis in the joint-space without considering the task-space; for example, principal component analysis [20]. In a recent work [21], Latash argues against such views on inter-joint coordination and synergy as synchronous joint rotations. To quote his words: “*we have to do better than measuring variables and performing correlation analysis.*”

This work is an effort to provide an effective analysis tool to study inter-joint coordination. To go beyond the limitations of model-free approaches (e.g., those based solely on correlation), we consider a model-based approach that accounts for the end-point movement as the goal of the kinematic chain. To this end, our approach is to exploit the *Inverse Kinematics* (IK) formulation from the robotic literature. More specifically, we use the weighted pseudo-inverse of the Jacobian matrix, where we identify the weight matrix from the observed kinematic data. These estimated weights serve as a quantification for the inter-joint coordination as they explain how much each joint participates in creating the end-effector velocity. This approach enables us to distinguish and quantify compensatory behavior in kinematic data when a baseline behavior is available. The body of research on IK formulation to study human kinematics has largely been overlooked using the weight matrix. This is not surprising since the estimation of these weights is not straightforward due to the nonlinear nature of the problem. In this work, we propose an algorithm to estimate such weights from the kinematics data. Furthermore, we show that such weights can be used to investigate the inter-joint coordination in rehabilitation scenarios as well as in prosthetic robotic arms applications.

## 2 Background

This section provides an overview of the related works from different areas of the literature. Subsection 2.1 reviews the weighted IK formulation in the robotic literature where it originated. The applications of this formulation and their related literature are categorized into two parts: analysis and control. In Subsection 2.2 reviews different analytical approaches for assessing movement quality, especially those which employ the IK formulation. Subsection 2.3 provides a review of possible applications where weighted IK is used for control purposes. Furthermore, Subsection 2.4 reviews possible applications which would benefit from a metric for inter-joint coordination; namely rehabilitation (e.g., for post-stroke patients), restoration (e.g., prosthetic robotic arms for individuals with amputation), and augmentation (e.g., supernumerary arms with the aim to assist a human-user). Finally, Subsection 2.5 provides mathematical background and formulates the problem of identifying the IK weights.

### 2.1 Weighted Inverse Kinematics formulation

In order to control a robotic manipulator, one needs to deal with *Forward* and *Inverse Kinematics*. Forward kinematics refers to the problem of determining the end-effector position when the joint values are known. This is often a simple operation when the geometrical properties of the chain are known. Inverse kinematics, however, aims at finding the joint values based on a given end-effector position. This problem becomes more challenging in redundant systems; i.e., having more degrees of freedom than the dimension of the task. In this case, one has to choose one particular IK solution among infinite possibilities. The solution to the IK problem can be categorized into two groups: closed-form and numerical solutions [22]. Closed-form solutions rely on geometrical properties of the kinematic chain or solve the IK problem in an algebraic form. However, numerical solutions are robot-independent but rely on different heuristics or iterative techniques. Furthermore, the IK problem can be addressed at the velocity level; i.e., finding the joint velocities based on a given end-effector velocity. In this case, it is helpful to differentiate the forward kinematics to obtain the Jacobian matrix, which maps joint velocities to the end-effector velocities at a given joint configuration. Thus, the pseudo-inverse of the Jacobian matrix can be used to solve the IK problem as initially formulated in [23]. A tremendous amount of research has been dedicated to overcoming this approach’s main limitation: dealing with singular configurations leading to a non-invertible Jacobian matrix or near-singular configurations resulting in large velocities. Several approaches to mitigate this problem can be found in the literature: cyclic coordinate descent [24], Levenberg-Marquardt damped least square [25], quasi-Newton and conjugate gradient [26], neural networks and artificial intelligence [27–29], Fuzzy inference [30], Genetic algorithms [31], Adaptive control using Jacobian transpose [32, 33], singular value decomposition [34–36], Damped Least squared [25, 37, 38], Quadratic programming [39]. Furthermore, using weighted pseudo-inverse for redundancy resolution is a common practice for robotic applications. Using weights (to pick a specific solution that minimizes a cost) was initially suggested in 1969 [40] and later implemented to control robotic manipulators [41, 42]. Most often, the manipulator inertia matrix is used as the weight matrix to minimize the total kinetic energy expenditure of the robot [43–45]. Nevertheless, in many robotic applications, the weight matrix choice might not directly affect the task performance [46]. In the same line, it is rare to see such approaches employed to analyze human movement. For instance, in [47] IK weights were used for modeling the kinematic data, which are manually set. In summary, the weighted IK formulation is less appreciated in the literature since there is no systematic method to choose a set of appropriate weights. Thus, researchers implicitly pick the identity matrix, retreating to the right pseudo-inverse, which is satisfactory for control purposes (as in [48]) but not sufficient for analysis.

### 2.2 The use of Inverse Kinematics in human movement analysis

Numerous metrics for movement quality have been proposed in the literature to analyze human motion; see [49] for a review. In the current state of the art, the kinematic measurements are often limited to the mean and standard deviation of joint motions and correlations analysis across joints; e.g., shoulder-elbow correlation. In [50], such kinematic measurements were used to study inter-joint coordination in post-stroke patients and compared to the clinical evaluation of the impairment by FMA-UE (Fugl-Meyer Assessment for Upper Extremity [51]). Studies using robotic-assisted protocols show that stroke patients exhibit abnormal intralimb joint coupling, which is correlated with FMA-UE [52–55]. Despite such a consensus, these clinical measures cannot capture small changes nor distinguish behavioral restitution from compensation. Other methods to study inter-joint coordination have been tried in the literature. For instance in [56, 57], inter-joint coordination is considered as the temporal relationship between the joint values. Similarly, in [58, 59], “continuous relative phase” has been used to study the effect of fatigue on inter-joint coordination. Such studies show that while these metrics are beneficial for assessment, they depend highly on the movement tasks. This is due to the fact that inter-joint coordination is often understood as correlation across joints without correcting for the fact that they are working toward a goal. Model-based approaches can help overcome these issues in investigating and quantifying inter-joint coordination.

The pseudo-inverse of the Jacobian matrix, as a model-based approach, is extensively used in the literature to analyze human kinematic data, mainly from a “motor-control” point of view. For instance, the well-known “Uncontrolled manifold” (UCM) method uses the Jacobian pseudo-inverse (more precisely, the null-space projector) to decompose the joint velocities into task-space and null-space [60, 61]. Null-space velocities (also referred to as “self-motion” [62]) are those which do not create an end-effector motion. By comparing the variations in the two spaces, the UCM method computes how strong the null-space is controlled (compared to the task-space). Thus, UCM views inter-joint coordination as how strong the joints contribute to the control of the null-space. Nevertheless, the same model-based approach using the pseudo-inverse of the Jacobian matrix can be used to quantify other facets of the inter-joint coordination; i.e., how much each joint contributes to creating an end-effector velocity. However, using an “a priori” assumed pseudo-inverse (right pseudo-inverse in this case) among infinite possibilities (i.e., weighted pseudo-inverse [63]) is an ill-posed approach to model kinematic data. Regardless of the choice for the pseudo-inverse, the modeling error (i.e., the part that cannot be explained by the pseudo-inverse) is attributed to the null-space. In summary, it can be seen that the problem of understanding the inter-joint coordination is the problem of finding the appropriate pseudo-inverse; i.e., finding IK weights that fit the data and rely less on the null-space.

### 2.3 The applications of weighted Inverse Kinematics in assistive robotics

Robotic-assisted rehabilitation has proven to be as effective as conventional training for upper and lower limb motor movement [64–68]. Moreover, robotic interventions provide two primary advantages. First, these devices (compared to traditional methods) allow for reliable measurements. Such measurements can be used to better analyze and track patient performance throughout the therapy. One challenge in this approach is determining the anatomical joint values based on the robotic ones. Many methods try to solve this problem by exploiting the IK formulation (e.g., [69]), which lies outside the scope of this work. The second advantage of such robotic systems is their capacity to influence patients’ kinematic behavior. This influence can be *active* where the robot assists the motion or *passive* where the robot acts as a damper and imposes a viscous field; i.e., active or passive from the robotic point of view. For instance, in a previous work [70], we used an upper-limb exoskeleton to modify the joint coordination by applying a force field in asymptomatic participants. Similarly, methods such as Constraint Induced Movement Therapy (CIMT) have been proposed where functional joints are restricted in order to encourage and rehabilitate the dysfunctional ones. Other works such as [70, 71] aim to alter the subject’s synergistic behavior using wearable robots. Even though rehabilitation robotics allows for various therapeutical approaches, the analysis of patients’ movements in terms of inter-joint coordination is limited. The use of pseudo-inverse methods in rehabilitation robotics is often criticized for 1) providing multiple solutions, 2) generating unnatural postures, and 3) lack of solution when the Jacobian matrix is singular. Therefore, there is an inclination toward closed-form solutions for rehabilitative applications [72]; for example, see [73–80]. While these analytical models provide reliable IK solutions for rehabilitation purposes, they cannot be used to measure the quality of the motion or to explain human kinematic data and its variability; e.g., individual differences or the evolution of the subject’s performance in the course of the therapy. This begs for modeling approaches to kinematics data where there are free and interpretable parameters such as the weights in weighted pseudo-inverse of the Jacobian matrix.

### 2.4 From inter-joint to human-robot coordination

Besides robotic-assisted rehabilitation, the weighted pseudo-inverse approach is of particular interest for assistive robotics; especially with the emerging cobots, robotic prosthetics, and robotic supernumerary arms. The resulting knowledge about human inter-joint coordination helps roboticists to design better robotic systems; e.g., humanoid robots, which move, interact and assist in a human-like manner. More specifically, the new generation of assistive robots requires systematic and efficient tools for analyzing and designing human-robot coordination. This is a necessary step toward ergonomic and efficient execution of daily tasks such as reaching motion across all domains: rehabilitation (e.g., in stroke patients), restoration (e.g., prosthetic for individuals with amputation), and augmentation (e.g., supernumerary arms to enhance industrial operators). In our previous works [81, 82], we used the concept of compensatory behavior to propose a new control paradigm for prosthetic arms; i.e., “Compensation Cancellation Control” as a movement-based strategy in contrast with EMG-based methods. Furthermore, in a recent work [83], we proposed a prosthetic control strategy specifically using the weighted pseudo-inverse of the Jacobian matrix. In such scenarios, a metric for inter-joint coordination can help the designer to assess the performance of a robotic assistive device. In other words, one could quantify how much the robotic joint contributes to the final end-effector movement. This view can be extended to any leader-follower robotic system with kinematic redundancies when the objective is to achieve higher assistive performance; i.e., higher follower’s contribution. Supernumerary robotic arms are also examples of such systems [84–86] where the human users use their proximal joints to compensate for the lack of robot’s activity. In all these examples, we are facing a similar question: ““how do we know if the robot/follower contributes enough to the task and not the user/leader compensating for the lack of robotic activity?”. In this work, we show that the weighted IK formulation can quantitatively answer such questions about human-robot coordination.

### 2.5 Mathematical background and problem formulation

Let us consider the observation of joint velocities ($\dot{q}\in R^{n}$ where *n* is the number of degrees of freedom) and end-effector velocities ($\dot{x}\in R^{m}$ where *m* is task/end-effector space dimension). Due to the geometry of the kinematic chain, joint velocities are mapped onto the end-effector velocities as:

$$\begin{array}{c} \dot{x}=J\left(q\right)\dot{q} \end{array}$$ (1)

which is known as *Forward Kinematics* with $J(q)\in R^{m\times n}$ as the Jacobian matrix. Let us note that the Jacobian matrix depends on the joint configuration ($q\in R^{n}$). However, the mapping from end-effector to joint velocities (i.e., *IK*) can be modeled as follows.

$$\begin{array}{c} \dot{q}=J^{\#}\dot{x}+{\dot{q}}_{n} \end{array}$$ (2)

where *JJ*^#^ *J* = *J* and $J{\dot{q}}_{n}=0$. It is trivial to show that the resulting $\dot{q}$ from any pair of *J*^#^ and ${\dot{q}}_{n}$ who satisfies these two conditions satisfies the forward kinematics in Eq 1. In this IK formulation, $J^{\#}\in R^{n\times m}$ is the *generalized inverse* of *J* (also called *Moore–Penrose inverse*). Furthermore, ${\dot{q}}_{n}$ represents the null-space velocities since it does not affect the end-effector velocities. Given a particular *J*^#^, the corresponding null-space velocity can be computed as:

$$\begin{array}{c} {\dot{q}}_{n}=N\dot{q} \end{array}$$ (3)

where *N* = *I*−*J*^#^ *J* is the null-space projector of *J*^#^. A practical and general choice for the *J*^#^ is the *weighted pseudo-inverse* [63]:

$$\begin{array}{c} J^{\#}=W^{-1}J^{T}{\left(JW^{-1}J^{T}\right)}^{-1} \end{array}$$ (4)

where $W\in {\text{R}}^{n\times n}$ is a positive definite matrix. This can be seen as the solution to the following problem:

$$\begin{array}{c} \begin{array}{cccc} & \underset{\dot{q}}{\text{min}} & & {\dot{q}}^{T}W\dot{q}\\ & \text{s.t.} & & J\dot{q}=\dot{x} \end{array} \end{array}$$ (5)

A diagonal *W* suffices in most applications as it already provides enough flexibility for design and analysis purposes. Moreover, multiplying *W* by a scalar does not affect Eq 4. Therefore, *W* can always be normalized by its largest element, leading to the following choice for this matrix:

$$\begin{array}{c} W=[\begin{array}{cccc} w_{1} & 0 & \cdots & 0\\ 0 & w_{2} & \cdots & 0\\ \vdots & \vdots & \ddots & \vdots \\ 0 & 0 & \cdots & w_{n} \end{array}]\;\;\text{where}\;w_{i}\in \left[0,1\right]\;\text{for}\;i=1...n \end{array}$$ (6)

Therefore, our goal is to estimate such weights (*w*_1_…*w*_*n*_) from the observed kinematic data ($\dot{q}$, $\dot{x}$, and *J*). However, it is essential to note that any choice of *W* can model the observed data. In other words, each choice of *W* brings us to a specific decomposition of observed joint velocities as follows

$$\begin{array}{c} \dot{q}={\dot{q}}_{task}+{\dot{q}}_{null} \end{array}$$ (7)

where ${\dot{q}}_{task}=J^{\#}\dot{x}$ and ${\dot{q}}_{null}=\dot{q}-{\dot{q}}_{task}$ which can be written as follows as well.

$$\begin{array}{cc} {\dot{q}}_{null} & =\dot{q}-{\dot{q}}_{task} \end{array}$$ (8)

$$\begin{array}{cc} & =\dot{q}-J^{\#}\dot{x} \end{array}$$ (9)

$$\begin{array}{cc} & =\dot{q}-J^{\#}J\dot{q} \end{array}$$ (10)

$$\begin{array}{cc} & =\left(I-J^{\#}J\right)\dot{q} \end{array}$$ (11)

$$\begin{array}{cc} & =N\dot{q} \end{array}$$ (12)

To illustrate the result of decomposition under different assumptions of *W*, we consider the following simple example of a redundant robot made of two serial prismatic joints with a one-dimensional task; i.e., *n* = 2 and *m* = 1. Let us consider an observed $\dot{q}={\left[0,1\right]}^{T}$ with *J* = [1, 1] which leads to $\dot{x}=1$. Using *W* = *I* to consider similar cost/contribution for each joint, we have *J*^#^ = [.5,.5]^*T*^, which leads to the following decomposition between task-space and null-space:

$$\begin{array}{c} {\dot{x}}_{task}=[\begin{array}{c} .5\\ .5 \end{array}]\;\text{and}\;{\dot{x}}_{null}=[\begin{array}{c} -.5\\ .5 \end{array}] \end{array}$$ (13)

While using *W* = diag([1, 1/9]), we have *J*^#^ = [.1,.9]^*T*^ with the following decomposition:

$$\begin{array}{c} {\dot{x}}_{task}=[\begin{array}{c} .1\\ .9 \end{array}]\;\text{and}\;{\dot{x}}_{null}=[\begin{array}{c} -.1\\ .1 \end{array}] \end{array}$$ (14)

The difference between the two cases reflects the underlying assumption about the IK strategy. In the first case, we assume that the two joints (being redundant) will contribute equally to the task (i.e., 0.5 each). However, the fact that the first joint has zero velocity is explained by the null-space; i.e., −.5 for the first joint and 0.5 for the second joint. In the second case, we assume a higher gain/cost for the first joint, which explains the observed velocity as mostly task-related (${\dot{q}}_{task}≃\dot{q}$) with comparatively less utilization of the null-space.

This simple example reflects our philosophy in modeling the joint velocities observed in a redundant system: **The observed joint velocities $\dot{q}$ need to be mostly explained by ${\dot{q}}_{task}$ where we correct for the imbalances of the contributions of joints by using a proper weight matrix.** As seen above, uniform joint contribution puts a strong assumption on IK strategy, which is not in line with the fact that joints in kinematically redundant systems have different roles, functionality, costs, and contribution. Therefore, by not correcting for such imbalances, we risk having a decomposition that is not descriptive of the actual underlying strategy for redundancy management. To summarize, it is important to make a distinction between the two following questions:

- How is the redundancy/abundancy utilized? (modeling the IK strategy)
- How is the geometrical null-space utilized? (velocity decomposition into task-space and null-space)

In our view, it is essential to begin by answering the first question by precisely modeling the kinematic data; e.g., in our case, estimating the IK weights. Only after answering the first question (i.e., finding the proper null-space projector) can it be examined how the null-space is utilized.

This work explains the observed kinematic behavior by estimating a proper set of IK weights. These weights can be interpreted as the relative cost of the joints as described in Eq 5; i.e., joints with lower/higher weights are less/more costly to move. These weights, therefore, explain inter-joint coordination in terms of joints’ contributions to creating the end-effector velocity.

## 3 Materials and methods

This section proposes an algorithm for estimating IK weights (Subsection 3.1) and our approach for numerical and experimental validation validation (Subsection 3.2).

### 3.1 Proposed algorithm for estimating Inverse Kinematics weights

Let us begin with a single observation at time-step *k* with ${\dot{q}}_{k}\in R^{n}$ as the joint velocities, ${\dot{x}}_{k}\in R^{m}$ as the end-effector velocities, and $J_{k}\in R^{m\times n}$ as the Jacobian matrix. We assume that this observation is generated by the following IK model:

$$\begin{array}{c} {\dot{q}}_{k}=J_{k}^{\#}{\dot{x}}_{k}+v_{k} \end{array}$$ (15)

where $J_{k}^{\#}=W^{-1}J_{k}^{T}{\left(J_{k}W^{-1}J_{k}^{T}\right)}^{-1}$ is the weighted pseudo-inverse of the Jacobian (*J*_*k*_) with the diagonal weight matrix $W\in R^{n\times n}$. In this model, *v*_*k*_ denotes the null-space velocities which do not affect the task space; i.e., *J*_*k*_ *v*_*k*_ = 0. However, our estimation process is formulated as follows.

$$\begin{array}{c} \left\{ \begin{array}{cc} {\dot{\bar{q}}}_{k} & ={\bar{J}}_{k}^{\#}{\dot{x}}_{k}+{\bar{v}}_{k}\\ {\bar{J}}_{k}^{\#} & ={\bar{W}}^{-1}J_{k}^{T}{\left(J_{k}{\bar{W}}^{-1}J_{k}^{T}\right)}^{-1}\\ {\bar{v}}_{k} & =\gamma \left(I-{\bar{J}}_{k}^{\#}J_{k}\right){\dot{q}}_{k}\\ e_{k} & ={\dot{q}}_{k}-{\dot{\bar{q}}}_{k} \end{array}\right. \end{array}$$ (16)

where $\bar{W}$, ${\bar{J}}^{\#}$, $\dot{\bar{q}}$, and $\bar{v}$ represent the estimations for their respective variables. To estimate the null-space velocities ${\bar{v}}_{k}$, we use the current estimation of the null-space projector; i.e., $\left(I-{\bar{J}}_{k}^{\#}J\right)$. However, we use 0 ≤ *γ* ≤ 1 as the “null-space projection ratio” to consider a portion of the projected velocities. The effect of *γ* becomes clear when inspecting the modeling error *e*_*k*_ which can be rewritten using Eq 16 and ${\dot{x}}_{k}=J_{k}{\dot{q}}_{k}$ as:

$$\begin{array}{c} e_{k}=\left(1-\gamma \right)\left(I-J^{\#}J\right){\dot{q}}_{k} \end{array}$$ (17)

which shows that there is no modeling error when entirely relying on the null-space projector; i.e., *e*_*k*_ = 0 for *γ* = 1. In other words, any weight matrix can perfectly model the data; for example, $\bar{W}=I$. This issue is further explained in the S1 File However, with smaller ratios, the error depends on the choice of $\bar{W}$. This allows us to determine a weight matrix that explains the underlying IK strategy rather than exploiting the null-space as a means for modeling error minimization. On the other hand, a small *γ* might lead to an undesirable estimation as it seeks to explain the null-space velocities using only the task-space part of the model.

**Algorithm 1:** Identification of *W* from observed data for *n* joints, *m* task dimensions, and *K* samples.

 **input**: $\dot{Q}\in {\text{R}}^{n\times K}$, $\dot{X}\in {\text{R}}^{m\times K}$, and $\{J_{1},J_{2},\ldots ,J_{K}\}\in {\text{R}}^{m\times n\times K}$

 **parameter**: *γ*

 **output**: $\tilde{w}$

1 Initialize $\tilde{w}=1_{n\times 1}$ and $\bar{V}=O_{n\times K}$;

2 **while** *e* > *ϵ* **do**

3  $\bar{W}\leftarrow \text{diag}\left(\tilde{w}\right)$;

4  *e* = 0;

5  **for** *k* ← 1,…, *K* **do**

6   ${\bar{J}}_{k}^{\#}={\bar{W}}^{-1}J_{k}^{T}{\left(J_{k}{\bar{W}}^{-1}J_{k}^{T}\right)}^{-1}$;

7   ${\dot{\bar{q}}}_{k}={\bar{J}}_{k}^{\#}{\dot{x}}_{k}+{\bar{v}}_{k}$;

8   $e\leftarrow e+\left|\right|{\dot{q}}_{k}-{\dot{\bar{q}}}_{k}\left|\right|$;

9   ${\bar{u}}_{k}=J_{k}^{T}{\left(J{\bar{W}}^{-1}J_{k}^{T}\right)}^{-1}{\dot{x}}_{k}$;

10   ${\bar{v}}_{k}=\gamma \;\left(I-{\bar{J}}_{k}^{\#}J_{k}\right){\dot{q}}_{k}$;

11  **end**

12  *e* ← *e*/*K*;

13  $\bar{U}=\left[{\bar{u}}_{1},{\bar{u}}_{2},...,{\bar{u}}_{K}\right]$;

14  $\bar{\Psi }=\left(\dot{Q}-\bar{V}\right){\dot{Q}}^{T}$;

15  $\bar{\Omega }=\bar{U}{\dot{Q}}^{T}$;

16  Rearrange $\bar{\Psi }\in {\text{R}}^{n\times n}$ into $\tilde{\Psi }\in {\text{R}}^{n^{2}\times n}$;

17  Rearrange $\bar{\Omega }\in {\text{R}}^{n\times n}$ into $\tilde{\Omega }\in {\text{R}}^{n^{2}\times 1}$;

18  $H={\tilde{\Psi }}^{T}\tilde{\Psi }$;

19  $F=-{\tilde{\Omega }}^{T}\tilde{\Psi }$;

20  $\tilde{w}\leftarrow$ Solve the *SQP* problem with *H* and *F* as in Eq 24;

21  $\tilde{w}\leftarrow \tilde{w}/\text{max}\left(\tilde{w}\right)$;

22 **end**

Algorithm 1 provides a heuristic to compute IK weights from such observed data. We assume that there are collected samples from a kinematic chain at each time-step *k* = 1,…, *K* which serve as the input to our algorithm; namely, the joint positions $\dot{Q}=\left[{\dot{q}}_{1},{\dot{q}}_{2},...,{\dot{q}}_{K}\right]\in R^{n\times K}$, end-effector velocities $\dot{X}=\left[{\dot{x}}_{1},{\dot{x}}_{2},...,{\dot{x}}_{K}\right]\in R^{m\times K}$, and $J_{k}\in R^{m\times n}$ as the Jacobian matrix at time-step *k* = 1…*K*. We initialize with $\bar{W}=I$ and zero null-space velocities $\bar{V}=0$ where $\bar{V}=\left[{\bar{v}}_{1},{\bar{v}}_{2},...,{\bar{v}}_{k}\right]\in R^{n\times K}$. In this algorithm, lines 1–9 correspond to Eq 16 where we also introduce an auxiliary variable ${\bar{u}}_{k}$ since we can write (from Eq 16):

$$\begin{array}{c} \bar{W}\left({\dot{\bar{q}}}_{k}-{\bar{v}}_{k}\right)=J_{k}^{T}{\left(J_{k}{\bar{W}}^{-1}J_{k}^{T}\right)}^{-1}{\dot{x}}_{k} \end{array}$$ (18)

where we define ${\bar{u}}_{k}=J_{k}^{T}{\left(J_{k}{\bar{W}}^{-1}J_{k}^{T}\right)}^{-1}{\dot{x}}_{k}$ for *k*th time-step and in the matrix form as $\bar{U}=\left[{\bar{u}}_{1},{\bar{u}}_{2},...,{\bar{u}}_{K}\right]\in R^{n\times K}$. Therefore, we can write Eq 18 in the matrix form as:

$$\begin{array}{c} \bar{W}\left(\dot{Q}-\bar{V}\right)=\bar{U} \end{array}$$ (19)

where we have the unknown variables as the linear coefficients, even though *U* implicitly depends on *W* in a nonlinear fashion. Furthermore, we can multiply both sides with ${\dot{Q}}^{T}$ and reach

$$\begin{array}{c} \bar{W}\bar{\Psi }=\bar{\Omega } \end{array}$$ (20)

where $\bar{\Psi }=\left(\dot{Q}-\bar{V}\right){\dot{Q}}^{T}\in R^{n\times n}$ and $\bar{\Omega }=\bar{U}{\dot{Q}}^{T}\in R^{n\times n}$. In this formulation, both $\bar{\Psi }$ and $\bar{\Omega }$ depend on our current estimation ($\bar{W}$). However, we can use our current estimation of $\bar{\Psi }$ and $\bar{\Omega }$ and update our estimation for $\bar{W}$ in a quasi-static manner as:

$$\begin{array}{c} {\bar{W}}_{t+1}\bar{\Psi }=\bar{\Omega } \end{array}$$ (21)

where ${\bar{W}}_{t}$ denotes our estimation at *t*th iteration. Since $\bar{W}$ is a diagonal matrix, we can arrange $\bar{\Psi }$ and $\bar{\Omega }$ regarding their columns as follows:

$$\begin{array}{c} \tilde{\Psi }{\tilde{w}}_{t+1}=\tilde{\Omega } \end{array}$$ (22)

where $\tilde{\Psi }={\left[diag\left({\bar{\Psi }}_{1}\right),diag\left({\bar{\Psi }}_{2}\right),...,diag\left({\bar{\Psi }}_{n}\right)\right]}^{T}\in R^{n^{2}\times n}$ where ${\bar{\Psi }}_{j}$ is the *j*^*th*^ column of $\bar{\Psi }$. similarly, $\tilde{\Omega }={\left[{\tilde{\Omega }}_{1}^{T},{\tilde{\Omega }}_{2}^{T},...,{\tilde{\Omega }}_{n}^{T}\right]}^{T}\in R^{n^{2}\times 1}$, and $\tilde{w}\in R^{n\times 1}$. In the S1 File, we show that Eq 22 is equivalent to quasi-newton methods.

At this stage, one might consider using least-square methods to solve for ${\tilde{w}}_{t+1}$, but there is no guarantee that the resulting values are positive. For this reason, we use Quadratic Programming in order to satisfy such constraints. To do this, we consider the following quadratic cost:

$$\begin{array}{c} C=\frac{1}{2}{\left(\tilde{\Psi }{\tilde{w}}_{t+1}-{\tilde{\Omega }}_{i}\right)}^{T}\left(\tilde{\Psi }{\tilde{w}}_{t+1}-\tilde{\Omega }\right) \end{array}$$ (23)

which can be simplified into:

$$\begin{array}{c} C=\frac{1}{2}{\tilde{w}}_{t+1}^{T}H{\tilde{w}}_{t+1}+F^{T}{\tilde{w}}_{t+1} \end{array}$$ (24)

where $H={\tilde{\Psi }}^{T}\tilde{\Psi }$ and $F=-{\tilde{\Omega }}^{T}\tilde{\Psi }$. We solve the QP with lower and upper bound constraints for the solution; i.e., to have each weight between zero and one. Finally, we normalize the weights in order to have the biggest weight at 1.

Our algorithm has two parts: evaluation (lines 2–11) and update (lines 12–20). While possible to swap the two parts, having the evaluation first allows us to have the performance of our initial guess for W. Since, in this work we start with *W* = *I*, we have the comparison of our result with the conventional *W* = *I* used in the literature. Finally, we stop the algorithm when the average norm-2 error is smaller than a designated threshold (line 2).

### 3.2 Experimental and numerical validation

To validate our proposed algorithm, first, we consider two low-dimensional simulations. The main purpose of these simulations is to illustrate the overall behavior of the proposed algorithm in terms of convergence to the nominal IK weights. The further details and the results of these simulations are reported in Subsection 4.1.

For the experimental validation, we consider kinematic data recorded in two different scenarios in the context of robotic-assisted rehabilitation and upper-limb prosthetic robotics, respectively. These studies were approved by the ethics committee of the Paris Descartes University (CERES—IRB number 20162000001072) and the Sorbonne University (SU CER-2021–111), respectively. All participants gave written and informed consent before participation. Further details and the results of these investigations are presented in Subsections 4.2 and 4.3.

## 4 Results

This section begins with two low-dimensional examples which illustrate the performance of the proposed algorithm in terms of convergence under different choices of parameters. Then, we report the results of two experimental use case scenarios for the proposed method, in which we identify the IK weights from the collected kinematic data. In both cases, we deal with human participants performing reaching motions with their upper extremities. In the first experiment, we use a robotic upper-limb exoskeleton applying a viscous force field to induce different kinematic behaviors. In the second experiment, we use a virtual prosthetic elbow with different control strategies. In terms of redundancies, this first case studies a 4 DoF kinematic chain for a 3d task, while the second experiment deals with a 3 DoF chain for a 2d task.

### 4.1 Illustrative examples

**Fig 1:**
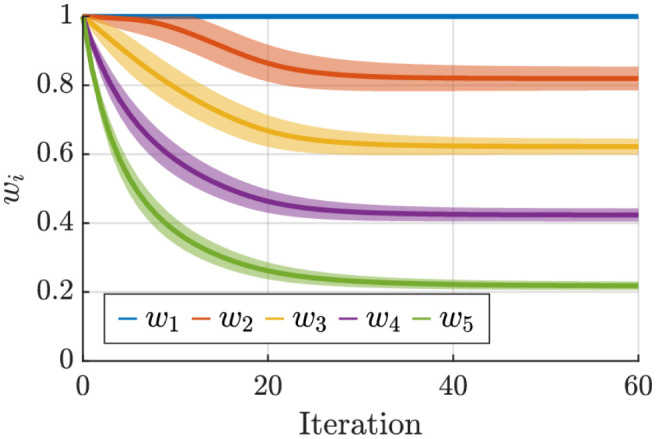
The convergence behavior of our proposed algorithm for randomly synthesized kinematic data. All five parameters converge to their respective optimal value. To obtain the standard deviation, we repeated the process 100 times. The average norm of the final error is 0.07.

In the first example, we provide a numerical evaluation in which all inputs to our algorithm are randomly generated (i.e., independent and identically distributed) using normal distributions. This means that all elements in $\dot{Q}$ and *J*_*k*_ are sampled from N(0,1) while *V* is sampled from N(0,σ) which allows us to study the effect of null-space velocity later. We consider *n* = 5 and *m* = 3 with *K* = 500 data points with w* = [1, 0.8, 0.6, 0.4, 0.2]^*T*^. For QP-solver, we set optimality, constraints, and step tolerances to 1*e*−3. We use *γ* = 0.6 and *σ* = 0.2. The results are illustrated in Fig 1 where the parameters converge to their respective optimal values. The final error is due to the null-space velocities. We show in the S1 File that a higher level of randomness in the null-space (*σ*) leads to a higher final error.

**Fig 2:**
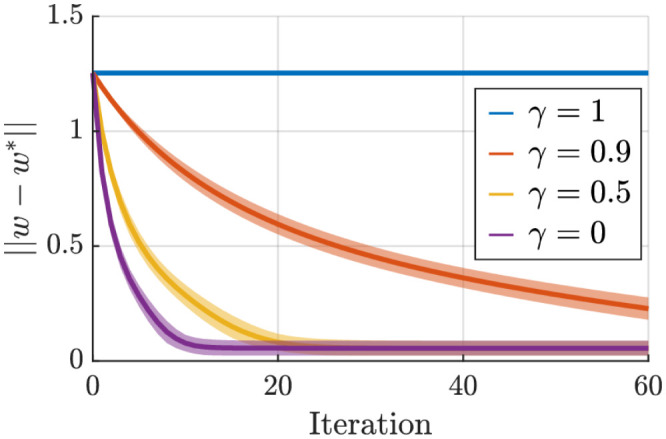
Visualization of the convergence behavior for different values of *γ* obtained from the random data. Since the null-space and task-space velocities are decorrelated, *γ* = 0 provides the fastest convergence.

Fig 2 studies the effect of *γ* on the convergence behavior. These results suggest that *γ* should be kept as low as possible. However, we should note that in this simulation, the null-space velocities *v*_*k*_ and ${\dot{q}}_{k}$ are uncorrelated (as they are generated in an iid manner). In the next simulation, we see that *γ* acts differently in practical and realistic scenarios since null-space and joint velocities are correlated. Nevertheless, this simulation confirms that *γ* = 1 does not lead to any weight update as analytically investigated earlier.

**Fig 3:**
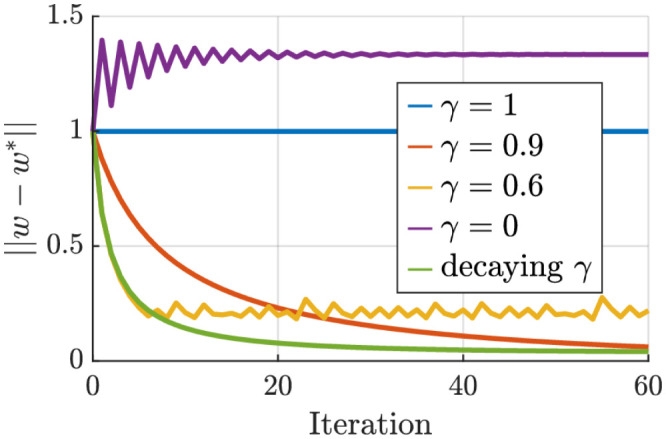
Visualization of the convergence behavior for different values of *γ* obtained from the two joints case.

In a second simulation, we consider a redundant kinematic chain with two joints and a one-dimensional task; i.e., *n* = 2 and *m* = 1. For the nominal IK solution, we consider *w*_1_ = 1 and *w*_2_ = 0.01 where we expect the task will be achieved by the second joint. Furthermore, for the null-space behavior, we consider *v*_*k*_ = [−0.1, *q*_*k*1_, 0]^*T*^ where we expect the first joint to be at zero. We simulate this system for 6*s* with *dt* = 0.1 starting from the initial position of *q*_1_ = [0.5, 0.5]^*T*^. This provides us with *K* = 60 data points. For the desired end-effector position, we use *x*_*g*_ = 1, then at *t* = 2 we switch to 1.5 and at *t* = 3 to 0.5. The results of our algorithm are illustrated in Fig 3. In this case, having *γ* > 0.5 is necessary for satisfactory convergence of the parameters. It is also interesting to see that, when the null-space velocities are not considered (*γ* = 0), the algorithm mistakenly converges to *w*_1_ = 0 and *w*_2_ = 1; i.e., describing that the task is accomplished mainly by the first joint, and the resulting discrepancy (between observed and expected joint velocities) is due to the null-space velocities. On the other hand, choosing higher values for *γ* leads to convergence to the optimal weights but at a lower convergence speed. In this manner, *γ* = 1 can be seen as a particular case where the convergence time is infinite. One way to deal with this trade-off is to use a time-varying ratio. For example, in this simulation, we tested *γ*_0_ = 0.6 with *γ*_*t*+ 1_ = *γ*_*t*_+ 0.6(1−*γ*_*t*_) in order to benefit from the fast convergence of *γ* = 6 at the beginning, and the optimality of a high *γ* toward the end. Generally, there is no procedure for choosing an optimal *γ* since the results depend on the dataset, as we see from the two last simulations. A helpful rule of thumb is to start with *γ* = 0.9; i.e., sacrificing convergence speed for optimality.

### 4.2 Experiment 1—Inter-joint coordination in a viscous field

**Fig 4:**
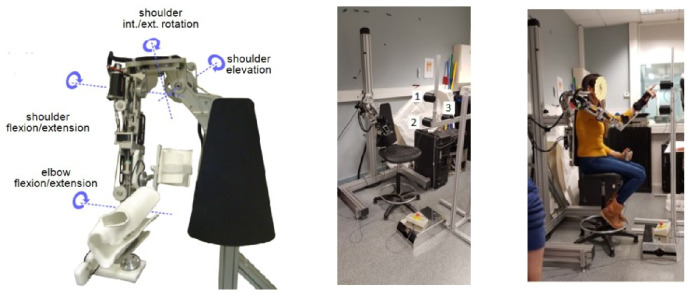
The experimental setup. The ABLE exoskeleton imposes a viscous field on the subjects, forcing them to change their kinematic behavior.

We collected kinematic data from 17 asymptomatic participants (11 males and 6 females), reaching three different targets. Each target was reached in 20 trials (some trials had to be removed due to recording issues) under two conditions: “Natural” and “Viscous”. As shown in Fig 4, the participants were wearing a shoulder-elbow exoskeleton; i.e., 4-DOF ABLE upper-limb exoskeleton [87]. This device allows us to impose a viscous force field (as presented in our previous work [70]) to modify the subjects’ inter-joint coordination. The ABLE exoskeleton offers quasi-static gravity and friction compensation with highly reversible mechanical transmission. However, due to a lack of dynamic compensation, the device exhibits a certain level of undesired resistance which is characterized in our previous work [88]. Nevertheless, this factor remains a constant effect across the two conditions since the participants wore the exoskeleton during both conditions. Furthermore, the Wrist movements were blocked using a prefabricated orthosis. In this manner, the analysis is limited to a 4 DoF kinematic chain (matching the exoskeleton’s DOF where we can apply an arbitrary force field) performing a 3 DoF task, leaving a redundant DoF.

Participants performed pointing movements towards three targets (1: high, 2: forward, 3: inward) with adjusted distance and height for each participant. The final orientation was not specified nor constrained, leading to a 3-dimensional task. The joint rotations were expressed according to the ABLE kinematics:

- ***q*_1_**: shoulder elevation around an anteroposterior axis.
- ***q*_2_**: shoulder axial rotation.
- ***q*_3_**: shoulder flexion in the plane defined by the two previous angles
- ***q*_4_**: elbow rotation.

Positive values indicate elevation, internal rotation, shoulder flexion, and elbow flexion. Finally, the mechanical joint limits of the exoskeleton were never reached by the participants during the reaching movement.

**Fig 5:**
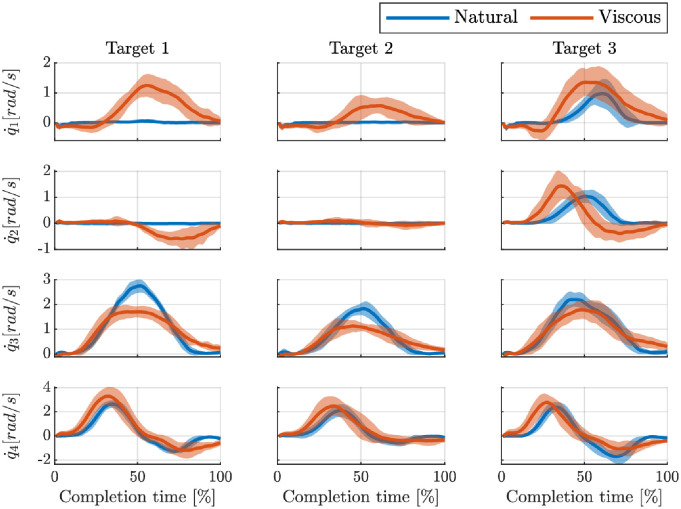
Comparison of a participant’s joint velocities when reaching the three targets in the two different conditions. The shaded areas represent the standard deviations.

Fig 5 shows the joint velocities of one of the participants reaching for the different targets. Here, we present the average velocities over trials, with the standard deviation as the shaded areas. Some observations can already be done at this stage. In the “Natural” Condition (i.e., transparent robot), the task is achieved mainly by utilizing the third and fourth joints (shoulder and elbow flexions), except for the third target, which requires the contribution of all joints. The main visible difference between “Natural” and “Viscous” is the higher utilization of the shoulder abduction. Moreover, these plots suggest that this participant has higher motor variability in the “Viscous” condition; i.e., larger shaded areas, especially for the first joint.

**Fig 6:**
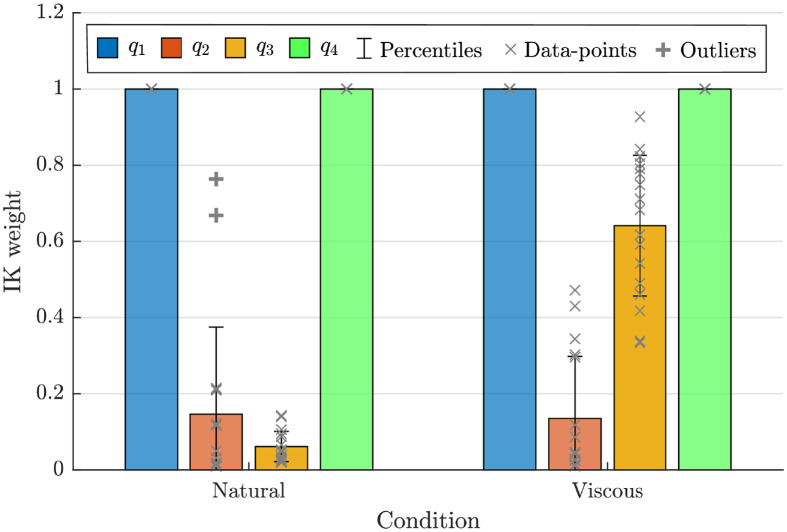
The estimated IK weights for the 17 participants in the two different conditions. The multivariate T-test (Hotling) shows a significant difference between the two set of the IK weights; *T*2 = 225.3, *F*(2, 15) = 105.6, and *p* = 0.000.

Fig 6 shows the result of our estimation algorithm for the IK weights of all the participants in the two conditions. In both conditions, the weights for the first and fourth joint are equal to one. As we presented earlier, we always scale the weights in order to have the largest value at 1. However, in this experimental setup, the fourth joint is over-specified; i.e., the effect of the fourth joint on end-effector velocity cannot be written as a linear combination of the other three joints. Therefore, for the IK, it does not matter what weight the fourth has. However, as a convention, we set this weight back to one right after solving QP, and we normalize only the first three first weights.

The estimated IK weights in the “Natural” condition show that, in a redundant configuration, the participants utilize the second and third joints over the first one. However, in the “Viscous” condition, the weight of the third joint significantly increases. This means the third joint is less used; consequently, other joints contribute more to the task. The resulting difference in IK weights (in Fig 6) provides a clear distinction between the two conditions; a distinction which is not easy to quantify from the trajectories shown in Fig 5, even though the qualitative difference is visible to the naked eye.

**Fig 7:**
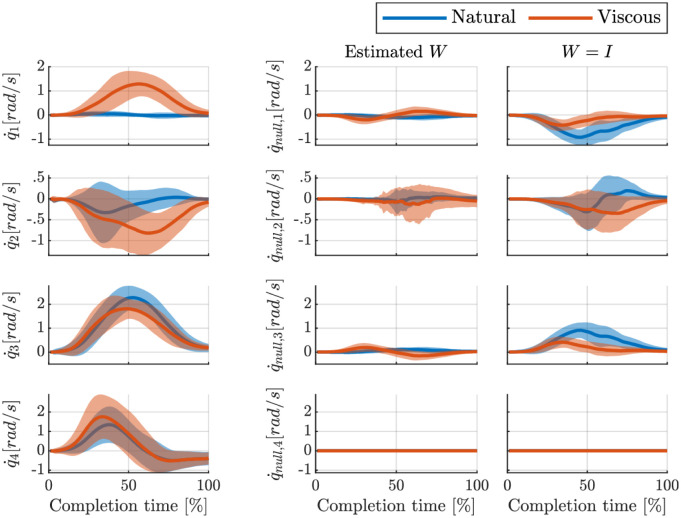
Extracted null-space velocities based on two different assumptions about the weight matrix. The first column shows the average velocity profiles over all subjects for Target 1. The Second column shows the extracted null-space velocity using the estimated weight matrix for each participant. The third column illustrates the null-space velocity when *W* = *I* is used.

To go further, we can look at the extracted null-space velocity under the estimated IK weights. These velocities are illustrated in Fig 7 which is averaged over all participants for the first reaching target. Moreover, we compare our extracted null-space velocities with the case where the identity matrix is used; i.e., *W* = *I* assuming equal contributions for all joints. Our results show that a major portion of the data is already explained by estimating the proper weights; i.e., the extracted null-space velocities are smaller compared to the case with *W* = *I*. Moreover, the “Viscous” condition has a higher level of null-space velocities (with a higher variance). However, *W* = *I* shows the opposite; in the “Natural” condition, humans utilize their null-space more with a higher variation. These results show that analyses over the null-space velocities computed with inappropriate weights can be deceptive.

### 4.3 Experiment 2—Human-robot inter-joint coordination

**Fig 8:**
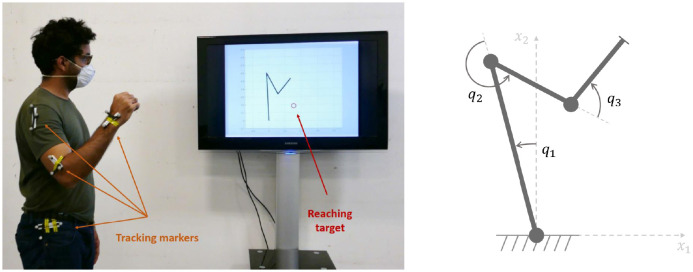
(Left) The second experimental setup where a human subject controls a virtual kinematic chain on the screen using respective bodily joints. (Right) A 3-link planar chain was used in this experiment. The first two joints (*q*_1_ and *q*_2_) are directly controlled using the participant’ hip and shoulder, respectively. The virtual elbow (*q*_3_) is controlled differently depending on the experimental condition. This figure is borrowed from our previous work [83] where we provide further technical details on the experimental setup and the control modes for the virtual elbow.

In this section, we examine an experimental case where the goal is to restore a natural IK strategy (i.e., joint movement) using a robotic prosthetic arm with different control modes. The proposed IK weights identification method was used to analyze the IK strategy obtained with the different control modes and identify the one restoring natural IK strategy. To this end, we asked participants to control a 3-DOF planar kinematic chain shown on a display screen using their upper body as shown in Fig 8; i.e., the three degrees of freedom on the screen correspond to the rotation of the hip (*q*_1_), shoulder (*q*_2_), and elbow (*q*_3_) in the sagittal plane. Using marker-clusters attached to the participants upper body, we tracked and transferred their body movements ([*q*_1_, *q*_2_, *q*_3_]) to a virtual kinematic chain displayed on a screen ($\left[{\bar{q}}_{1},{\bar{q}}_{2},{\bar{q}}_{3}\right]$). While we directly mapped the hip and the shoulder, different mappings from the participant’s elbow to the virtual one (${\bar{q}}_{3}$) were used in each condition. Here, we detail these conditions:

- **Natural**: The elbow is directly mapped. Thus, the participant has full/direct control over the virtual kinematic chain; i.e., ${\dot{\bar{q}}}_{3}={\dot{q}}_{3}$.
- **Locked**: The virtual elbow is locked at a specific angle; i.e., ${\dot{\bar{q}}}_{3}=0$. Thus, the participant has partial control over the virtual chain.
- **Coupled**: The virtual elbow velocity is coupled to the participant shoulder velocity; ${\dot{\bar{q}}}_{3}={\dot{q}}_{2}$. Thus, the participant has partial but indirect control over the virtual elbow.
- **Assistive**: The virtual elbow is controlled based on our previously proposed method in [83]. Thus, the participant has partial and indirect control.

In each condition, the participants reached for a sequence of target positions; i.e., 28 different targets, each displayed for 6s. We performed this experiment with 10 participants.

The controller used in the **Assistive** condition can be summarized as follows:

$$\begin{array}{c} \left\{ \begin{array}{c} {\dot{\bar{q}}}_{3}=J_{3}^{\#}v-N_{3}K\left(q-\hat{q}\right)\\ \dot{v}=-\alpha \left(v-\dot{x}\right) \end{array}\right. \end{array}$$ (25)

where $J_{3}^{\#}$ and *N*_3_ are the third rows of *J*^#^ and *N*, respectively. *J*^#^ is computed as in Eq 4 with *W* = *diag*([1, 1,.15]). To cancel the compensatory role of the hip and the shoulder, we use *K* = *diag*([2,.2, 0]), and $\hat{q}=\left[0,-2.7,0\right]$ *rad*. *v* can be considered as the low-pass filter version of the end-effector velocity $\dot{x}$ with *α* = 40. In simple words, this proposed method amplifies at the end-effector level the velocities the human subject creates by the natural joints while helping the user with the posture. This controller can be seen as a nonlinear task-dependent synergy approach; i.e., a nonlinear mapping from ${\dot{q}}_{1}$ and ${\dot{q}}_{2}$ to ${\dot{\bar{q}}}_{3}$. During this mode, the position of the virtual elbow (*q*_3_) was limited to [0, *π*] rad.

**Fig 9:**
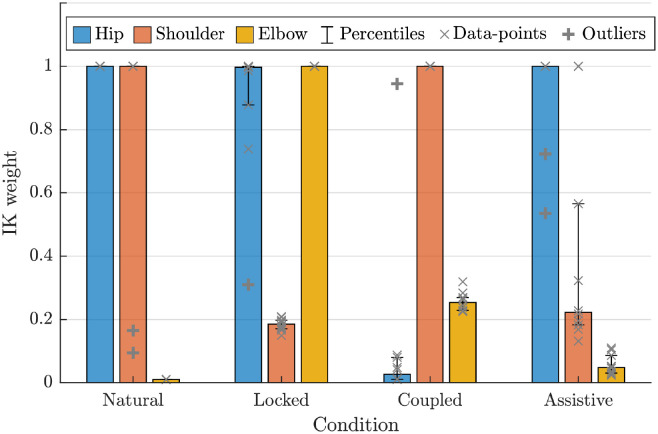
The estimated IK weights for the 10 subjects in the four different conditions.

The results for the estimated IK weights are presented in Fig 9. For the first condition (“Natural”), we estimate high weights for the hip and shoulder but a low one for the elbow. This means the human participants prefer to use the elbow joint even if the same end-effector velocity can be created using other joints. The result for the second condition (“Locked”) is straightforward. In this case, the virtual elbow is not contributing to the end-effector velocities, thus a high IK weight. Moreover, if the same end-effector velocity can be created by the hip or the shoulder, the participants use the shoulder; therefore, a lower weight for the shoulder. In the third condition (“Coupled”), we have a low IK weight for the hip. This shows that the participants tend to use the hip when it comes to hip-shoulder or hip-elbow redundancy. Furthermore, comparing the IK weight of the elbow to the shoulder reveals that such simple synergy methods are only effective locally; i.e., the elbow contributes more than the shoulder, while the hip compensates for the lack of contribution of both shoulder and elbow.

In the last condition (“Assistive”), we have satisfactory IK weights as the joints contribute more when moving up the kinematic chain (proximal to distal). Compared to the Natural case, we have a high weight for the hip (i.e., compensation cancellation) and low weight for the elbow (i.e., proactivity of the prosthetic joint). Thus, the main difference is the behavior of the shoulder joint. This means that in shoulder-elbow redundancy, the elbow is not fully taking over the shoulder as in the Natural condition. This result is expected given the nature of decentralized control in leader-follower setups; i.e., the natural joints need to initiate the desired end-effector velocity as a means for intention communication. In other words, the elbow joint cannot assist until it observes an end-effector velocity generated by the hip or the shoulder.

This result shows that our proposed estimation algorithm is capable of pinpointing the differences across conditions where different control strategies are used for a prosthetic joint. The resulting IK weights are interpretable (given the IK formulation) and coherent with the settings in each condition. Moreover, the resulting IK weight favors our assistive controller as it leads to a set of estimated IK weights that are qualitatively closer to the “Natural” condition. This result corroborates our previous findings where this type of assistive strategy leads to lower hip utilization [83]. Hip-utilization is an intuitive metric since the human participants tend to perform the 2D task mostly with the shoulder and elbow joints in the “Natural” condition. The participants begin to use the hip joint as compensation when they lose direct control over the virtual elbow joint; i.e., using the hip and the shoulder joint. Therefore, in this experiment, the inter-joint coordination can be interpreted as non-using the hip joint. In general cases, the objective of a prosthetic joint can be formulated similarly; i.e., obtaining a “naturalistic” inter-joint coordination or IK behavior which can be quantified using our proposed estimation algorithm. Finally, it is also interesting to note that we use weighted IK formulation in our proposed controller. As discussed in [83], these nominal weights characterize the dynamics and subsequently influence the system’s performance. However, it is important to note that the estimated weights are not only a result of the underlying control mechanism but also the executed motions.

## 5 Discussion

We here proposed a data-driven approach to quantify inter-joint coordination using weighted pseudo-inverse of the Jacobian matrix. More specifically, we provided an estimation algorithm for these corresponding weights that are interpretable in light of Eq 5 as the respective cost of each joint. As a convention, we assumed these weights to be between zero and one, with the most costly joint always at 1. Such scaling is possible since only the relative ratio of weights matters in Eq 4. However, the resulting weights might not be straightforward when we compare different conditions. For example, in Fig 6, the weight of the first joint is one while its ratio to the third joint changes. To overcome these issues, we can consider a transformation to represent the level of contribution rather than cost as follows:

$$\begin{array}{c} \beta_{i}=\frac{w_{i}^{-1}}{\sum_{k}w_{k}^{-1}} \end{array}$$ (26)

These “contribution coefficients” sum to one and can be easier to interpret across conditions. For example, for the first experiment we have *β* = [0.04, 0.27, 0.65, 0.04] for the natural condition, which changes to *β* = [0.09, 0.68, 0.14, 0.09] in the viscous condition. Here, it is quicker to notice that all joints increase their contribution coefficient to compensate for the third joint. The same transformation can be applied to the second experiment, which results in Table 1.

**Table 1:** Transformation of IK weights to “Contribution coefficients” for the second experiment.

Condition	Contribution coefficient		
	hip	shoulder	prosthetic elbow
Natural	0.01	0.01	0.98
Locked	0.15	0.72	0.13
Synergy	0.62	0.08	0.30
Assistive	0.05	0.12	0.83

In this work, as described in Eq 5, we assumed time-invariant state-independent weights for our IK formulation. For instance, it can be imagined that fatigue can change the IK strategy over time. Furthermore, our proposed metric to quantify inter-joint coordination is of “spatial” nature since it averages over all the data points across time. Therefore, this metric is not capable of pinpointing any temporal coordination. For instance, the two following conceptual scenarios lead to similar IK weights: 1) two joints equally contribute at all times, 2) the second joint contributes equally after the first joint is done with its contribution. To overcome this, one might consider estimating the IK weight for different time intervals of the motion. Comparing the estimated weights across intervals might provide insight into temporal coordination. However, increasing the number of intervals deteriorates the estimation performance; i.e., increasing the parameters-to-observations ratio. At its limit, we can imagine time-dependent IK weights, which will have the same dimensionality as the kinematic data; i.e., *n*×*T* when *n* is DoF and *T* the length of a trial. Thus, those time-dependent IK weights will appear as a nonlinear (and task-dependent) averaging of the trials, which might bring us closer to spatio-temporal inter-joint coordination. Even if such time-dependent weights are obtained, they will not directly provide a metric for “temporal” inter-joint coordination.

It is important to consider an essential caveat in interpreting the IK weights when measuring an assistive robot’s performance: not all contribution of a robotic joint to the end-effector velocity is a contribution toward the human-intended goal. Nevertheless, in such leader-follower setups, the human corrects (as much as possible) the disturbances that the robotic joint introduces. In turn, this corrective behavior of the human (which is done by utilizing the natural joints) affects IK weights for the natural joints; i.e., lower IK weights for the natural joints. Therefore, such human corrective behaviors provide robustness in analyzing the IK weights for leader-follower setups.

From a clinical point of view, the IK weights are essential to pinpoint and quantify the compensatory behaviors [16]. For example, IK weights could document the increased use of trunk flexion to compensate for shoulder-elbow impairment in stroke patients [15], or increased proximal motion in amputees wearing a prosthesis to compensate for the lack of wrist mobility [89–91]. Compensatory behavior is critical to document within the clinical assessment to follow the patients’ progress and establish a rehabilitation strategy. Therapy may reduce compensatory behavior to avoid “learned non-use” [92] or musculoskeletal disorders. Conversely, compensatory motions may be trained (skill learning) in order to improve daily life activities in stroke patients [93] or prosthetic use by amputees [89].

Finally, it is vital to note that this work takes an IK-based approach to only “*quantify*” the inter-joint coordination in kinematic data. This does not entail that the individuals (or the central nervous system) solve a weighted IK problem to create hand movements. Nevertheless, numerous works in the literature try to explain how the brain handles redundancies [9, 62, 94, 95]. Furthermore, we formulated our estimation problem at the kinematic level; i.e., ignoring the effect of inertia, gravity, and other dynamical aspects of human movement. Investigating inter-joint coordination at the level of dynamics might provide a better picture. However, it would be more cumbersome when we need to estimate the applied torques and the dynamic properties of the human arm. In such a formulation, we would try to explain how a required force at the end-effector maps onto joint torques. A similar approach has been explored in human movement studies [11] and widely used in robotic literature [96]. Nevertheless, the effect of dynamics (and other higher-level mechanisms such as neural mechanisms) are partially captured at the kinematics level; i.e., the estimated weights are influenced by the dynamic behavior since joint velocities can be seen as the results of joint torques. The same argument is applied to the joint limits as their effects are captured by the estimated weights. For instance, the lack of contribution of a joint that operates near its limit will be reflected as a higher IK weight.

The identified weights from human data could benefit assistive robotics and neurorehabilitation. One current challenge in rehabilitation robotics is the control of exoskeletons which has to be performed at the joint level; i.e., finding the appropriate *reference* joint trajectories which are human-like. While it can be relatively trivial to obtain reference trajectories for the end-effector [97], finding the corresponding joint trajectories is not straightforward due to the multiplicity of solutions that arise from the kinematic redundancies. To address these issues, oftentimes, trajectories are copied from previously recorded movements in healthy subjects, from recordings from therapists mimicking the reference movement, or are computed based on some inferred optimality principle of human movements. However, relying on such input limits the efficacy of the control algorithms because the resulting trajectories are generally expressed as time-dependent position values which do not generalize to different movements, targets, and tasks. This means that the patient’s freedom of movement with the exoskeleton is limited as coordinated patterns can only be programmed for specific movements [97]. Therefore, the proposed method could allow extracting IK weights from human data and offer a more generic tool and less restrictive approach to generating coordinated reference trajectories. In general, any collaborative robotic device could benefit from this identification method. For instance, a physically interactive cobot could use such identified weights to exhibit human-like kinematic behavior, improving the predictability of its user.

## 6 Conclusion

In conclusion, this work attempted to go beyond correlations analysis when quantifying inter-joint coordination. To this end, we considered a task-dependent formulation: i.e., the joints rotate to move the end-effector via the geometric coupling formulated by the Jacobian matrix. We showed that velocity decomposition into task and null-space highly depends on our choice for the IK weights. We argued that IK weights should be estimated in order to explain the observed velocities, mainly using the task-space. Based on this argument, we proposed an optimization algorithm where *γ* (the null-space projection ratio) plays an important role. To deal with the nonlinearities with respect to the weight matrix, we solved the problem in a quasi-static manner. Furthermore, we employed a quadratic programming formulation to respect the constraints of having positive weights. These resulting weights have clear interpretations: the joints with higher weights are more costly to be moved to create the desired end-effector velocity. This view on inter-joint coordination has substantial implications for clinical applications and robotic applications such as robotic prosthetic arms. We showed the result for two different experiments where the estimated weights are in line with experimental designs; i.e., pinpointing the effect of a viscous field over participant’s joint coordination in the first experiment and favoring a specific assistive controller for a prosthesis in the second experiment.

## Supporting information

S1 File (PDF)

S1 Dataset Kinematic recordings from Experiment 1. This dataset is provided as a Matlab Mat-file containing 17 cells, one per participant. Each cell is organized in a structure with *conditions*→*targets*→*trials* format, where there are two conditions (“Natural” and “Viscous”) and three targets (Target 1, 2, and 3). The number of trials for each target might vary as we removed those which had recording issues. Each trail provides joint position, joint velocities, and end-effector velocities. These quantities are stored in 100×4 matrices; 100 time-steps with *dt* = 0.009, and 4 degrees of freedom. Moreover, the Jacobian matrix for each time step can be found for each trial. Furthermore, in this dataset, we provide some of the quantities which are computed using the proposed algorithm; i.e., estimated IK weights for each participant, the estimated null-space velocities based on *W* = *I*, and estimated weights for each trial. Moreover, we provide the concatenated data (over targets and trials) for each condition as “IK_data” which is ready to be passed to the proposed algorithm.
(ZIP)

S2 Dataset Kinematic recordings from Experiment 2. This dataset is also provided as a Matlab Mat-file containing 10 cells, one per participant. Each cell contains the recordings for four different conditions: “Natural”, “Locked”, “Coupled”, and “Assistive”. In this dataset, there is a single trial for each condition which contains the time-vector, human joint values (3 DoF for hip, shoulder, and elbow), virtual elbow (1 DoF), end-effector position, and the position of displayed targets. Furthermore, we also provide the prepared data for the proposed algorithm as “IK_data” for each trial, along with the estimated IK weights.
(ZIP)

S1 Data Matlab implementation of the proposed algorithm. This source code (compressed as a zip file) provides a Matlab implementation of the proposed algorithm. The necessary instruction to run the source code is provided in “ReadMe.txt”.
(ZIP)
