# Supplementary material for: Identification of inverse kinematic parameters in redundant systems: Towards quantification of inter-joint coordination in the human upper extremity
Source: PLoS One. 2022 Dec 16;17(12):e0278228. doi: 10.1371/journal.pone.0278228 (PMC9757603; doi:10.1371/journal.pone.0278228)
Supplement: S1 File — (PDF) [file pone.0278228.s001.pdf]

S1 Appendix. Relation to Quasi-newton methods.
Let us consider the following cost function ($g : \mathbb{R}^n \mapsto \mathbb{R}^+$)

$$g(x) = \frac{1}{2}(Ax - f(x))^T (Ax - f(x)) \qquad (27)$$

where $x \in \mathbb{R}^n$, $A \in \mathbb{R}^{m \times n}$, and $f : \mathbb{R}^n \mapsto \mathbb{R}^m$. This cost function can be approximated as

$$g(x) \simeq g(x_n) + \nabla_g(x_n)^T (x - x_n) \qquad (28)$$

Therefore, a Newton step to find $g(x_{n+1}) = 0$ satisfies

$$g(x_n) + \nabla_g(x_n)^T (x_{n+1} - x_n) = 0 \qquad (29)$$

We approximate the gradient as follows

$$\nabla_g(x) \simeq \frac{1}{2} A^T (Ax - f(x)) \qquad (30)$$

where we neglected $\nabla_f(x)$ and a scaling factor of $\frac{1}{2}$ is applied. In this manner, we have

$$\frac{1}{2}(Ax_n - f(x_n))^T(Ax_n - f(x_n) + A(x_{n+1} - x_n)) = 0 \tag{31}$$

which simplifies to:

$$\frac{1}{2}(Ax_n - f(x_n))^T(Ax_{n+1} - f(x_n)) = 0 \tag{32}$$

Before converging to the zero ($Ax_n \neq f(x_n)$), the update rule should satisfy:

$$Ax_{n+1} = f(x_n) \tag{33}$$

Therefore such an update rule can be used to solve $Ax = f(x)$ as illustrated in Fig 10. We can see that the convergence is achieved only if the slope of the nonlinear part is smaller than the linear part; i.e., if $\nabla_f(x)$ can be neglected compared to A.

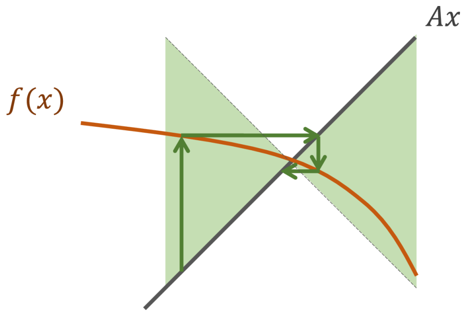

Fig 10. Illustration of an quasi-static approach to solve $Ax = f(x)$.

S2 Appendix. Effect of the null-space projector.
Here, we justify why the use of γ is necessary. In order words, we explain why removing the estimated null-space velocities is not practical. Let us assume at iteration i we have an estimation for weights as W_i, which leads to the pseudo-inverse $J_i^{\#}$. Therefore, our identification problem becomes:

$$\dot{q} = J_{i+1}^{\#}\dot{x} + (I - J_i^{\#}J)\dot{q} \tag{34}$$

where we are looking for the next set of weight (W_{i+1}) that leads to $J_{i+1}^{\#}$, which satisfies this equation. Using $\dot{x} = J\dot{q}$, we can rewrite this equation as

$$(I - J_{i+1}^{\#}J)\dot{q} = (I - J_i^{\#}J)\dot{q} \tag{35}$$

which trivially shows that the previous set of weights is a solution to this equation. Therefore, using a specific set of weights for the null-projector leads to identifying the same weights. This is observed in Fig 2 where $\gamma = 1$ leads to instant convergence (which could be sub-optimal). Furthermore, even in the case of $\gamma = 1$, we still perform one step of identification for W without removal of any null-space velocities; i.e., initialization $\bar{V} = 0$ in the first iteration. However, using the null-space of J (i.e., with $W = I$) to estimate and remove $\bar{V} = 0$ would lead to the wrong identification of $W = I$.

This fact is investigated in Fig 11 where we study the effect of σ and γ on final estimation error (after 100 iterations). We can see that higher variance in null-space velocities (higher σ) causes higher estimation error. Furthermore, γ seems not to have a considerable effect as long as it is below 0.8.

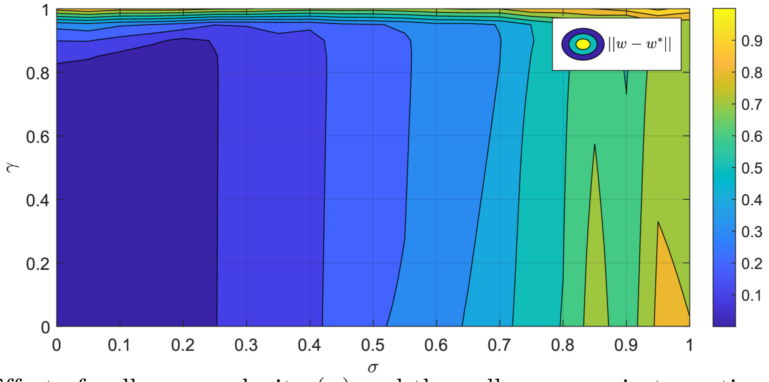

Fig 11. Effect of null-space velocity (σ) and the null-space projector ratio our (γ) on convergence error ($||w - w^*||$). The results are generated using a fully randomized dataset.

S3 Appendix. Orthogonality vs. de-correlation

Given a decomposition of $\dot{q}$, it is interesting to look at inter product and correlation between the parts; namely, $\dot{q}_{task}$ and $\dot{q}_{null}$.

$$\begin{aligned} \dot{q}_{task}^T \dot{q}_{null} &= \dot{x}^T J^{\#T}(I - J^{\#}J)\dot{q} \\ &= \dot{q}^T J^T J^{\#T}(I - J^{\#}J)\dot{q} \\ &= \dot{q}^T J^T (JW^{-1}J^T)^{-1} JW^{-1}(I - J^{\#}J)\dot{q} \end{aligned} \tag{36}$$

This shows that in the case of $W = I$, we have $J(I - J^{\#}J) = 0$ leading to an orthogonal decomposition. Furthermore, we can look at the cross-covariance between task-related and null-space velocities as follows:

$$\begin{aligned} K_{null,task} &= \mathbb{E}[(\dot{q}_{null} - \mathbb{E}[\dot{q}_{null}])(\dot{q}_{task} - \mathbb{E}[\dot{q}_{task}])^T] \\ &= \mathbb{E}[N(\dot{q} - \mathbb{E}[\dot{q}])(\dot{q} - \mathbb{E}[\dot{q}])^T J^T J^{\#T}] \\ &= \mathbb{E}[N(K_{\dot{q}} + D)J^T J^{\#T}] \end{aligned} \tag{37}$$

where $D = (\dot{q} - \mathbb{E}[\dot{q}])(\dot{q} - \mathbb{E}[\dot{q}])^T - K_{\dot{q}}$ with $\mathbb{E}[D] = 0$. We can also use the property that $NW^{-1}J^T J^{\#T} = 0$ (since $NJ^{\#} = 0$) to write:

$$K_{null,task} = \mathbb{E}[N(K_{\dot{q}} - \kappa W^{-1} + D)J^T J^{\#T}] \tag{38}$$

where $\kappa \in \mathbb{R}$ is an arbitrary scalar. This equation shows how the difference between data covariance ($K_{\dot{q}}$) and the assumed weight matrix (W) affects the cross-variance between the two parts. For instance, in the case where the variation of J over data points can be neglected (since J depends on q), we can reach:

$$\begin{aligned} K_{null,task} &= N(K_{\dot{q}} - \kappa W^{-1})J^T J^{\#T} + N\mathbb{E}[D]J^T J^{\#T} \\ &= N(K_{\dot{q}} - \kappa W^{-1})J^T J^{\#T} \end{aligned} \tag{39}$$

Therefore, with the choice $W = \kappa K_{\dot{q}}^{-1}$, the two parts are decorrelated. In general, this analysis shows that with a proper choice of W, one can minimize the correlation between that null-space and task-relevant velocities. Such a choice is linked to $K_{\dot{q}}^{-1}$ (the covariance of $\dot{q}$), which hints at having higher/lower IK weights for joints that are less/more varied. Nevertheless, in a realistic scenario, such a direct link with $K_{\dot{q}}$ does not exist since J changes over time and cannot be taken out of the expectation. In other words, due to the nonlinearity of the problem, the observed data ($\dot{q}$) is contaminated by

the Jacobian matrix J. Thus, one needs to utilize both pieces of information ($\dot{q}$ and J) as in our proposed algorithm to derive the IK weights.

Fig 12 shows the quality of the decomposition for the synthetic data in Fig 1. In this plot, the measure for correlation is $||K_{null,task}||$ and the measure for orthogonality is the average angle between $\dot{q}_{task}$ and $\dot{q}_{null}$. The values at zero iteration correspond to the initial value where $W = I$ with a 90deg angle at the expense of a high correlation between the null and task velocities. As the optimization progresses, we reach a lower level of correlation (down to 14%) by allowing 2 degrees of rotation. As seen here, one can sacrifice orthogonality for decorrelation. Such distinction is analogous to the difference between Principal Component Analysis (PCA) and Independent Component Analysis (ICA), where one aims at orthogonal components while the other looks for independent ones.

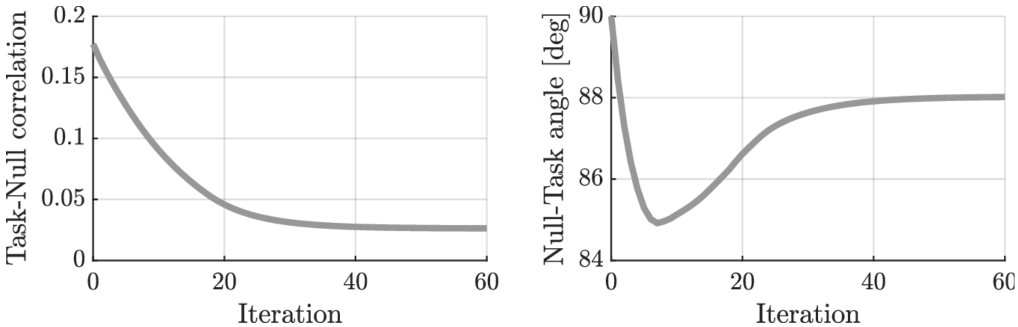

Fig 12. The quality of the velocity decomposition into task-space and null-spaces for the estimated IK weights in Fig 1 in terms of decorrelation and orthogonality. By slightly sacrificing the orthogonality (for 2det) the correlation between the two subspaces is reduced drastically (down to 14%).
